# Green Synthesis and the Evaluation of Osteogenic Potential of Novel Europium-Doped-Monetite Calcium Phosphate by Cissus quadrangularis

**DOI:** 10.7759/cureus.59202

**Published:** 2024-04-28

**Authors:** Nidhita Suresh, G Kaarthikeyan

**Affiliations:** 1 Department of Periodontics, Saveetha Dental College and Hospitals, Saveetha Institute of Medical and Technical Sciences, Saveetha University, Chennai, IND

**Keywords:** calcium phosphate, green synthesis, bone regeneration, monetite, europium

## Abstract

Background

The quest for an ideal bone grafting material has been ongoing for decades. Calcium phosphate, alone or in combination with other materials in natural bone, has been shown to aid in bone regeneration effectively. Monetite exhibits superior solubility and resorption rates among calcium phosphates, rendering it an optimal choice for bone regeneration applications. However, the degradation rate of the Monetite is much faster than that of all the other calcium phosphates. Hence, we have added Europium onto the matrix to alter the degradation profile and enhance the osteogenic ability of the prepared matrix.

Materials and methods

An exclusive Europium-Monetite composite was synthesized employing eco-friendly techniques involving *Cissus quadrangularis. *The osteogenic potential was gauged using the MG-63 cell line through a calcium mineralization assay employing an Alizarin Red solution, collagen estimation, and an alkaline phosphatase (ALP) assay. The composite's cytocompatibility was evaluated using the MTT (3-(4,5-dimethylthiazol-2-yl)-2,5-diphenyltetrazolium bromide) assay across different concentrations ranging from 12.5 µg to 100 µg.

Results

Scanning electron microscopy (SEM) analysis of the Europium-Monetite composite revealed a sheet-like arrangement in stacks, and the ATR-IR confirmed the presence of elements Ca, P, and Eu. The osteogenic potential, analyzed by ALP activity, calcium mineralization, and collagen staining, was 10% higher than that of the control (Monetite).

Conclusion

The prepared novel Europium-Monetite calcium phosphate complex can enhance the osteogenic potential and could be a promising material for bone regeneration/tissue engineering. The newly created Europium-Monetite calcium phosphate complex holds promise for various bone grafting applications, including integration into scaffolds and as a coating for implants.

## Introduction

Enormous progress has been made in bone regeneration to bring an alternative to autologous grafts, as autologous grafts are associated with disadvantages such as limited availability and morbidity to the patient. Numerous techniques have been used routinely with biomaterials such as allografts, xenografts, alloplastic, and growth factors. The gaps resulting from the utilization of alloplastic calcium phosphate grafts pose challenges including incomplete setting reactions leading to inflammation. Moreover, their lack of a macro-porous structure restricts cell adhesion speed, fluid exchange, and restoration capabilities. Additionally, they become brittle under tensile and shear stress [1].

Nevertheless, Monetite calcium phosphates used as bone grafts have demonstrated bone augmentation rates similar to those of autografts, which are considered the gold standard and sourced from the patient's own body [1]. They undergo conversion of the hydroxyapatite without any phase transformation. They also exhibit faster resorption and greater volume of newly formed bone [1]. However, the downside of using this Monetite as a bone augmentation material is its weak mechanical properties and efficacy in biological performance and degradation. Numerous studies have focused on improving the biological performance of Monetite structures by incorporating specific modifications into their matrix. Incorporating strontium into Monetite structures enhances the long-term viability of osteoblast-like cells, indicating potential applications in conditions characterized by excessive bone resorption [2,3]. Rare earth nano-biomaterials are booming in bone tissue engineering and implant design [4]. Europium is a rare earth element of the lanthanide series used initially in medical applications for imaging due to its fluorescent properties [5]. These materials have been used effectively for bone regeneration due to their unique osteogenic, angiogenic, antimicrobial, and antioxidant properties and in vivo bone tissue imaging [5]. Numerous physical and chemical methods have been mediated for the synthesis of nanoparticles. Green nanoparticle synthesis using plants is more advantageous as it can stabilize the agents in the nanoparticles and are nontoxic, eco-friendly, and sustainable [6-8]. *Cissus quadrangularis*, a vining plant indigenous to India and Africa, belongs to the Vitaceae family and is also known as *Vitis quadrangularis*. It has been utilized for medicinal purposes for centuries. The stem and root of this plant exhibit antioxidant and antimicrobial properties. *C. quadrangularis* is rich in anabolic steroidal compounds and significant amounts of calcium and phosphorus. *C. quadrangularis* functioned as a reducing agent in producing silver, ZnO, and CaO nanoparticles [9]. Hence, to improve their osteogenic potential in the present study, we have incorporated Europium into the Monetite calcium phosphate. Therefore, we have fabricated an innovative and novel Monetite-structured green synthesis of Europium-Monetite calcium phosphate by *C. quadrangularis*, and the material's physiochemical characterization and osteogenic potential were assessed. 

## Materials and methods

**Figure 1:**
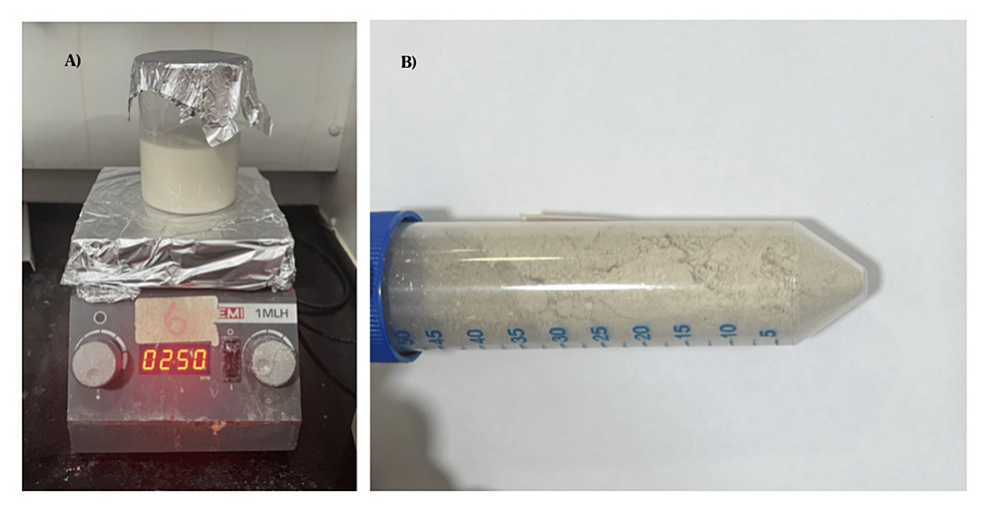
A shows the green synthesis of Europium-Monetite by C. Quadrangularis, and B shows the Europium-Monetite composite

The innovative Europium-Monetite composite was synthesized through green synthesis using *C. quadrangularis*, and its osteogenic potential was evaluated through calcium mineralization assay using Alizarin Red solution, collagen estimation, and alkaline phosphatase (ALP) assay. The ethical clearance has been successfully obtained from the institutional review board under reference SRB/SDC/PhD/PERIO-2312/23/TH-080. To prepare the Europium-Monetite composite via green synthesis using *C. quadrangularis*, 2 g of *C. quadrangularis* were dissolved in 100 mL of distilled water and incubated in a shaker overnight at 37°C as shown in Figure 1A. After filtration, the filtrate was combined with 0.99 mol of calcium nitrate solution and 0.01 mol of Europium, stirring for three to four hours. Then, 0.67 mol of diammonium hydrogen phosphate was introduced to the stirred solution, which was further stirred for 24 hours until reaching a pH of 7.0. The solution was subsequently dried as shown in Figure 1B yielding the sample for further analysis, including physiochemical characterization. 

SEM and EDX analysis

Scanning electron microscopy (SEM) was utilized to assess the physical properties, while X-ray diffraction (XRD) analysis was employed to examine mineral phases and crystallinity. The surface morphology and topography were evaluated using SEM, conducted with a high-energy beam and electrons' backscattering, with X-rays' characteristics recorded and converted into images by electron detectors (FESEM, JOEL JSM IT800 (JEOL Ltd., Tokyo, Japan)). Images were observed at various magnifications to analyze surface topography. Energy dispersive X-ray (EDX) analysis of the material was conducted using an EDX detector X-PLORE-30/C-SWIFT (Oxford Instruments, Wiesbaden, Germany) to determine the elemental composition correlated with the atomic number. This EDX analysis was coupled with SEM.

XPS analysis

X-ray photoelectron spectroscopy (XPS) analysis was performed using the Thermo Scientific instrument, Model NEXSA surface analysis. The instrument features a micro-focused monochromatic Al-Kα source (hν=1486.6 eV), a hemispherical analyzer, and a 128-channel plate detector. This analysis aimed to characterize the surface properties of the novel Europium-doped-Monetite calcium phosphate.

Raman spectroscopy

Raman spectroscopy provides insights into the molecular composition of the Europium-doped-Monetite calcium phosphate complex. Analyzing the spectrum can reveal information about the various molecules present. If its components are present, relative peak intensities offer quantitative data regarding the composition of the mixture. The Raman spectroscopy of the innovative Europium-Monetite complex (Eu-MCaP) was carried out using WITEC ALPHA300 RA-Confocal Raman (AFM Microscope, Ulm, Germany).

Cytotoxic assay

Biocompatibility tests were conducted using human osteoblastic-like cells (MG63) cultured in Dulbecco's Modified Eagle Medium (DMEM, Sigma Aldrich). The metabolic activity of the cells was assessed using the MTT (3-(4,5-dimethylthiazol-2-yl)-2,5-diphenyltetrazolium bromide) assay. Europium-doped-Monetite calcium phosphate samples at different concentrations were added to 96-well plates along with the cells, followed by a 24-hour incubation period. The assay was then measured using a microplate reader at a wavelength of 570 nm. Cell viability percentage was calculated using the formula: Cell viability (%) = (Test sample OD)_570 / (Control OD)_570 × 100

Calcium mineralization assay

Using Alizarin Red solution, a dye that binds to calcium salts, the calcium generation in the control and experimental group Eu-mCaP was assessed at seven days. Calcium content was evaluated using the Alizarin Red S (ARS) treated cells, where the cells would be incubated at room temperature for 20-30 min with 1 mL of 40 mM ARS per well. The cells were incubated for three days, washed with phosphate-buffered saline (PBS) then fixed with 4% formaldehyde at room temperature for 15 min. The samples were viewed under the fluorescent microscope (Leica Stellaris) and analyzed after washing the cells.

ARS-treated cells were mixed with 10% (v/v) acetic acid, agitated, and incubated for 30 min. The cells were then taken and placed in tubes, agitated for 30 sec, and incubated at 85°C for 10 min. Subsequently, it was placed for centrifugation for 15 min with 200 µL of supernatant and 10% NH_4_OH (v/v) of 22.5 µL 0.405 nm was used to measure the absorbance.

Collagen estimation

Osteoblasts produce the initial matrix consisting mostly of collagen after that the matrix is mineralized by the deposition of minerals. In this study, we evaluated the amount of collagen by staining the collagen using the histological method for control cells and cells incubated with Europium-Monetite calcium phosphate. Collagen estimation was performed by incubating the cells at 37°C for 48 hours with medium for both control and treated cells every 24 hours. After incubation, cells would be washed with saline, then harvested and fixed for 20 min with 4% formalin. Following fixation, the cells were rinsed with PBS solution three times and stained with 0.1% Sirius Red (20 μL) at 37°C for 20 min. After this, the cells were treated with 10% acetic acid and washed with PBS solution. Then these cells were stained with Picro-Sirius Red for one hour. The samples were visualized under a fluorescent microscope after washing with acidified water and dehydrated with ethanol. Two blind investigators, working independently, meticulously conducted quantitative estimations of histochemical stainings. Each investigator thoroughly analyzed all tissue specimens, and their respective findings were harmoniously averaged and visually depicted according to established criteria. This approach ensures robustness and reliability in our data interpretation.

ALP assay

The activity of ALP serves as an indicator of the osteogenic differentiation process from mesenchymal stem cells to osteoblasts. This enzyme, expressed by osteoblasts, plays a crucial role in biomineralization by increasing the concentration of inorganic phosphate (Pi) through adenosine triphosphate (ATP) hydrolysis. The deposition of calcium and phosphate ions marks the initial stage of extracellular matrix mineralization during bone formation.

ALP activity was assessed by measuring protein production. Cells were seeded with the sample and incubated for three days. Before incubation, cells were solubilized with Triton-X-100 and incubated for 1 min. Cell density was measured at 405 nm using an ELISA plate reader, and images were captured using a fluorescence microscope after adding 5-bromo-4-chloro-3-indolyl-phosphate (BCIP) and nitro blue tetrazolium (NBT) solution and incubating for 30 min. Protein content was determined using the Bradford assay. Samples were placed onto a new 96-well plate with Bradford reagent and AP buffer. Optical density was measured at each culture time by dividing the optical density by the cell count at 595 nm.

## Results

**Figure 2:**
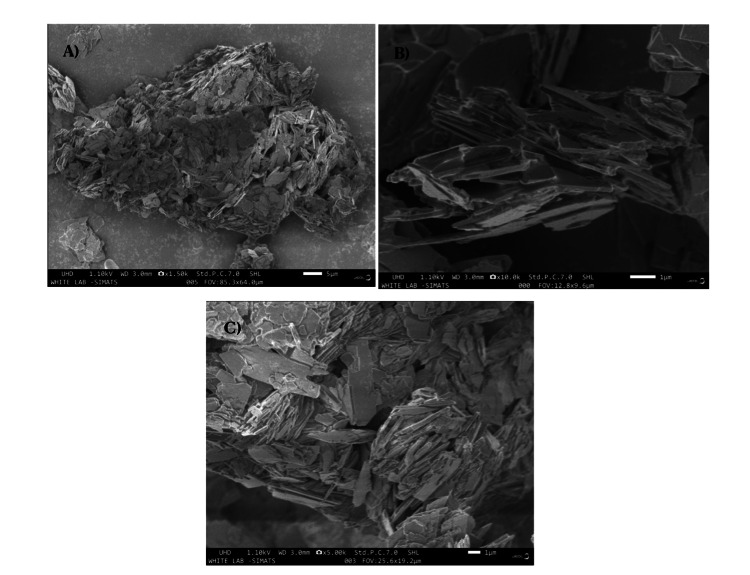
SEM Images of the green synthesis of Europium-doped-Monetite phase of calcium phosphate at A) x1.50k, B) x10.0k, and C) x5.00k SEM: scanning electron microscopy

**Figure 3:**
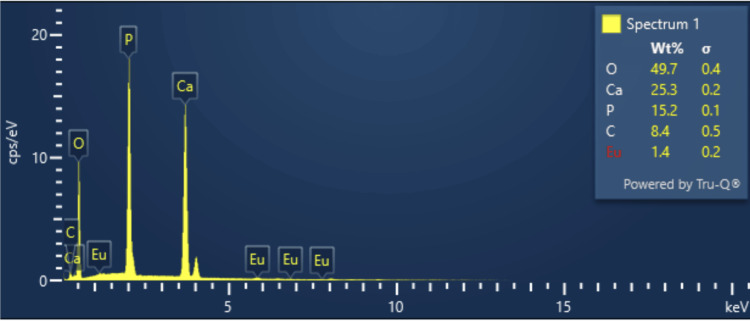
Elemental composition of the prepared compound by EDX analysis EDX: energy dispersive X-ray

**Figure 4:**
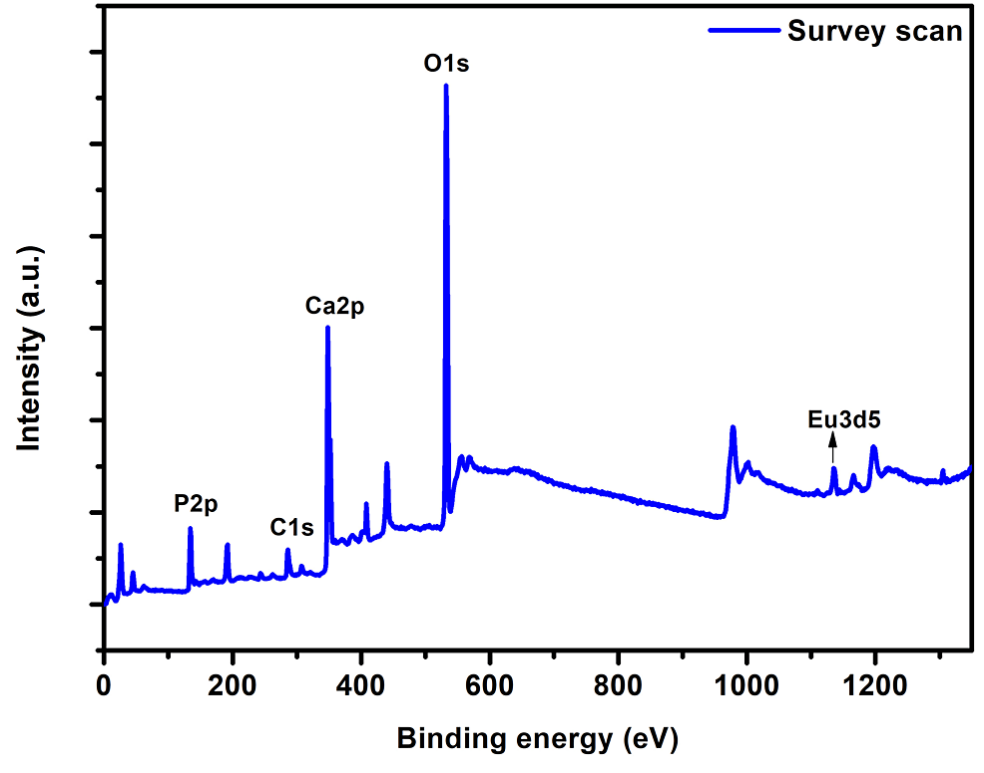
XPS spectra analysis for Europium-Monetite calcium phosphate XPS: X-ray photoelectron spectroscopy

**Figure 5:**
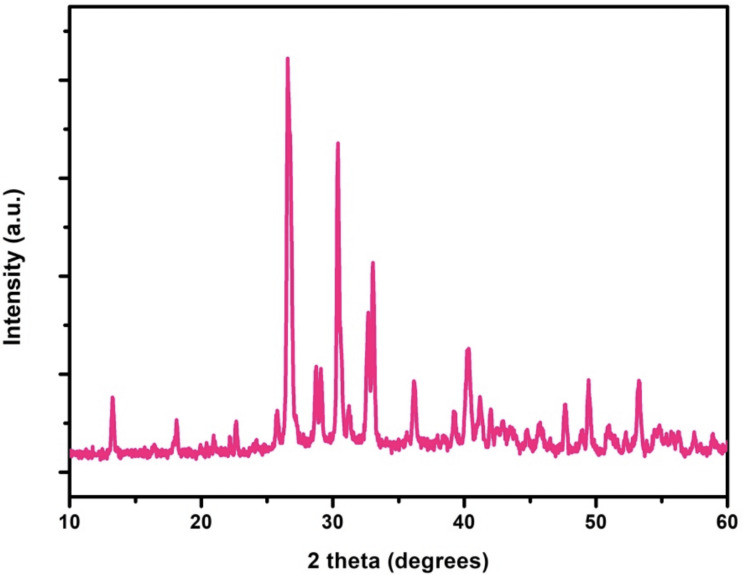
XRD spectrum of Europium-doped-Monetite phase of calcium phosphate XRD: X-ray diffraction

**Figure 6:**
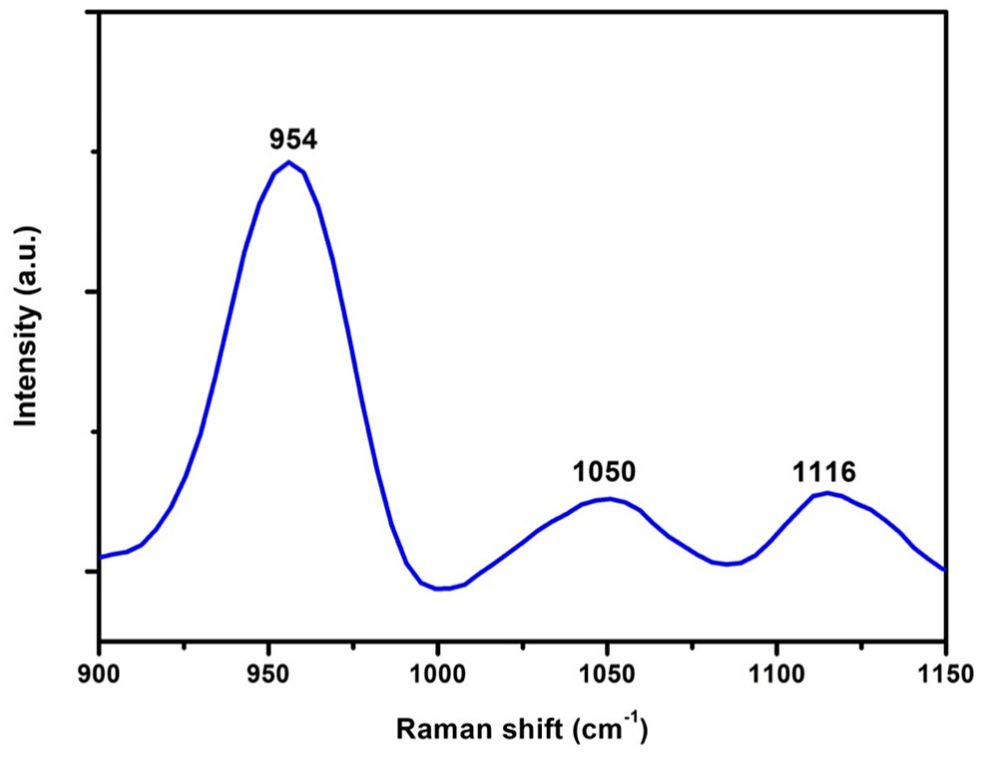
Intensity peaks observed by Raman spectrum

The surface topography of Monetite exhibited a sheet-like configuration arranged in stacks. SEM micrographs of various magnifications of x1.50k, x10.0k, and x5.0k are observed as shown in Figure 2. The composite featured a sheet-like arrangement with a thickness of 90 nm and a length of 1.2 µm. In spectrum 1, the weight percentages of the elements were 25.3%, 15.2%, and 1.4% for Ca, P, and Eu, and atomic percentages were 0.2%, 0.1%, and 0.2% for Ca, P, and Eu, respectively, as illustrated in Figure 3. XPS spectra analysis revealed intensity peaks of various elements in the Europium-doped-Monetite phase of calcium phosphate. It visually represents these peaks, including 133.9 for P2p, 351.5 for Ca2p, 531.7 for O1s, and 1135.5, 1165.2, and 1169.4 for Eu 3d5 as shown in Figure 4. The XRD spectrum obtained for the novel Europium-doped-Monetite calcium phosphate matches with JCPDS No. 98-000-556. The structure of Monetite Ca(HPO_4_) was determined to be anorthic and the peak between 20° and 30° depicts the Monetite as shown in Figure 5 and the thinner peak corresponds to the bigger crystal. Intensity peaks were observed by Raman spectroscopy at 954 cm21 corresponds to the n1 stretching of PO4 confirming the presence of carbonated apatite, 1050 cm^-1^ for HSO_4_-ions and 1116 cm^-1^ attributed to C-O stretching as shown in Figure 6 [8,9]. 

**Figure 7:**
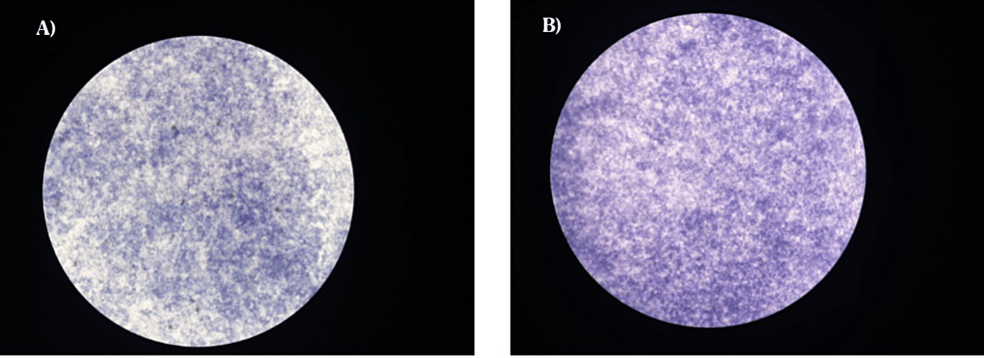
A shows ALP activity observed in the microscopic staining panels for control, and B shows Eu-MCaP ALP: alkaline phosphatase; Eu-MCaP: Europium-Monetite complex

**Figure 8:**
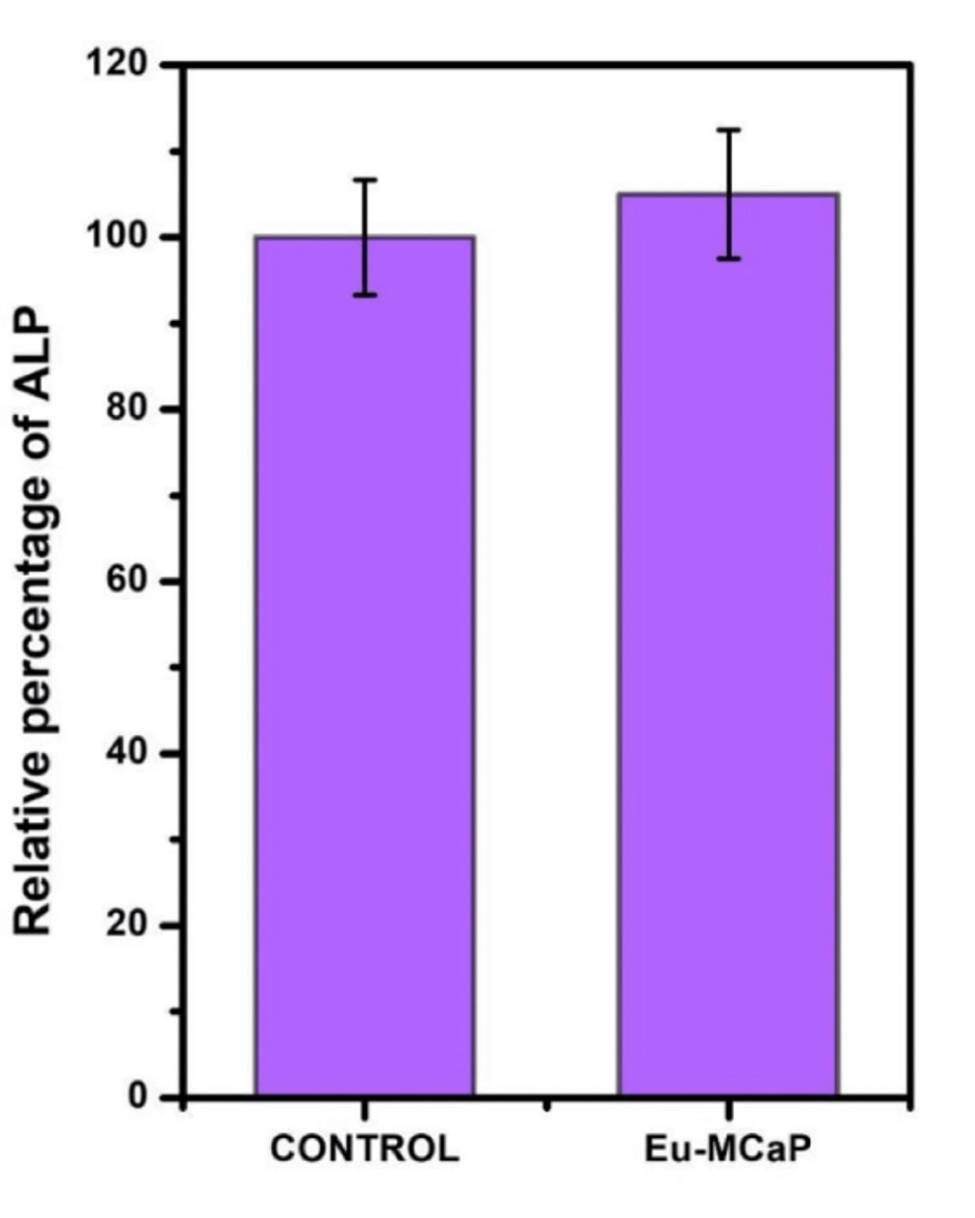
Relative percentage of ALP activity in control and Eu-MCaP ALP: alkaline phosphate; Eu-MCaP: Europium-Monetite complex

ALP assessment for cells incubated with Europium-Monetite calcium phosphate, compared to the control (cells without incubation), revealed significantly darker blue-violet staining. As confirmed through blue-violet staining, ALP-active cells exhibited higher levels of Europium-Monetite calcium phosphate than the control as shown in Figures 7A, 7B. The percentage of ALP activity in the control and Europium-Monetite calcium phosphate complex was tabulated in a bar graph as shown in Figure 8.

**Figure 9:**
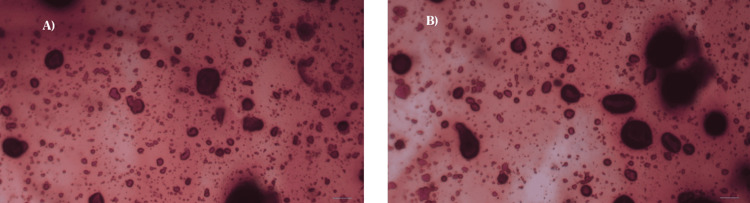
A shows calcium staining observed microscopically in control, and B shows calcium staining observed microscopically when incubated in Eu-MCaP Eu-MCaP: Europium-Monetite complex

**Figure 10:**
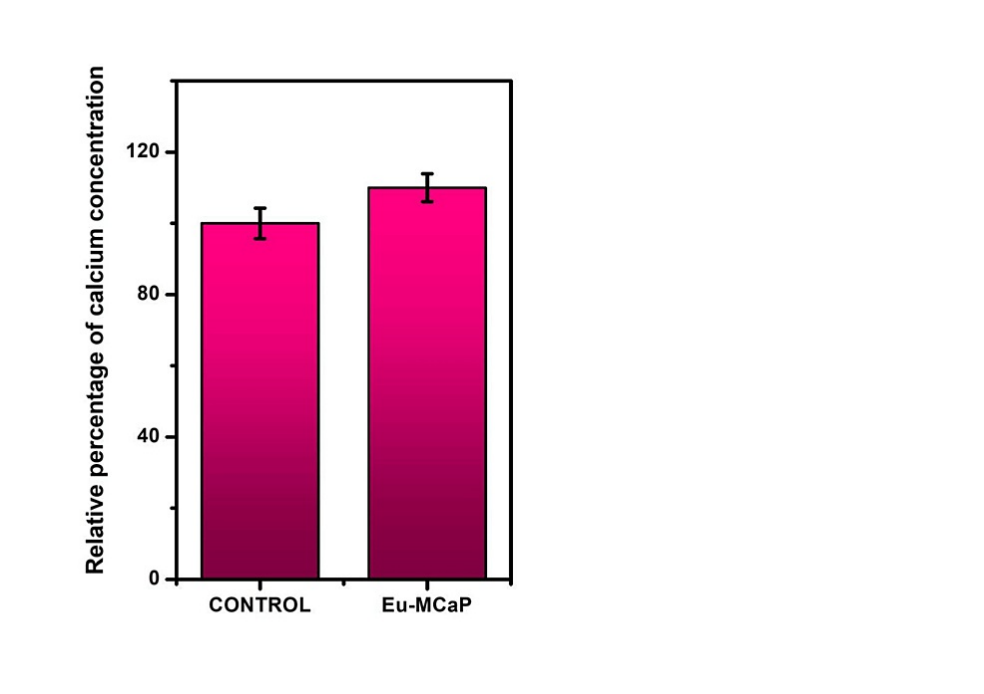
Relative percentage of calcium staining in control and Eu-MCaP Eu-MCaP: Europium-Monetite complex

Calcium-rich deposition on cells after incubation with Eu-MCaP was quantified using ARS staining. The Eu-MCaP composition, featuring calcium phosphate, facilitated the formation of calcium nodules on cells. ARS staining showed dense red spots in the experimental group, indicating the presence of calcium nodules promoting bone regeneration as shown in Figures 9A, 9B [10,11]. Eu-MCaP stimulated mature bone cells and precursors accelerated osteoblast activity through ERK1/2 and PI3K/Akt pathways and played a vital role in controlling osteoclast production and resorption [12,13]. The relative percentage of calcium staining was higher in the Europium-Monetite calcium phosphate group than in the control as shown in Figure 10.

**Figure 11:**
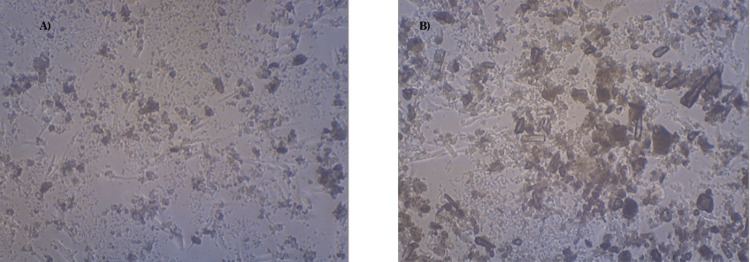
A shows collagen staining observed microscopically in control, and B shows collagen staining observed microscopically when incubated with Eu-MCaP Eu-MCaP: Europium-Monetite complex

**Figure 12:**
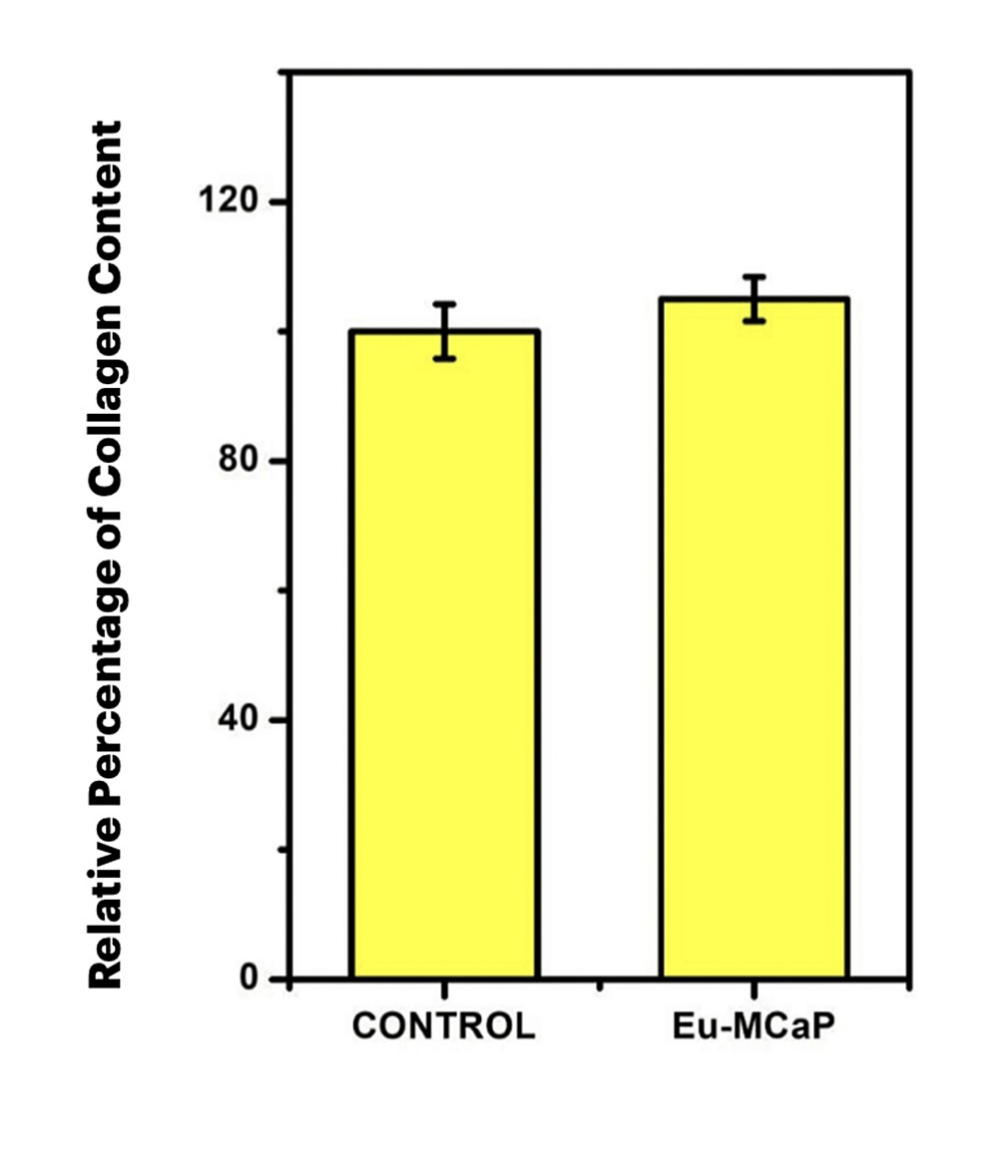
Relative percentage of collagen content between control and Eu-mCaP Eu-mCaP: Europium-Monetite complex

Collagen matrix production, assessed by collagen staining during incubation, demonstrated higher quantities for Eu-MCaP than control cells, showcasing the material's efficacy in enhancing bone regeneration as shown in Figures 11A, 11B. The relative percentage of collagen staining was higher for the Eu-MCaP than the control and was tabulated in a bar graph as shown in Figure 12.

**Figure 13:**
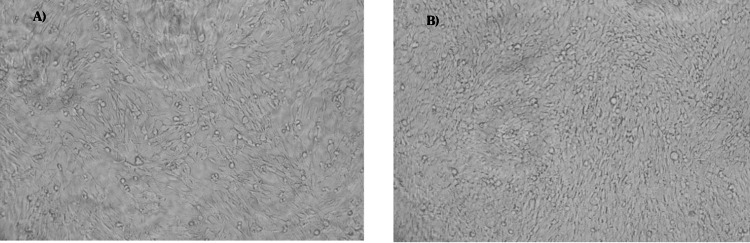
A shows cell viability observed microscopically in control, and B shows cell viability observed microscopically at 100 µg Eu-MCaP Eu-MCaP: Europium-Monetite complex

**Figure 14:**
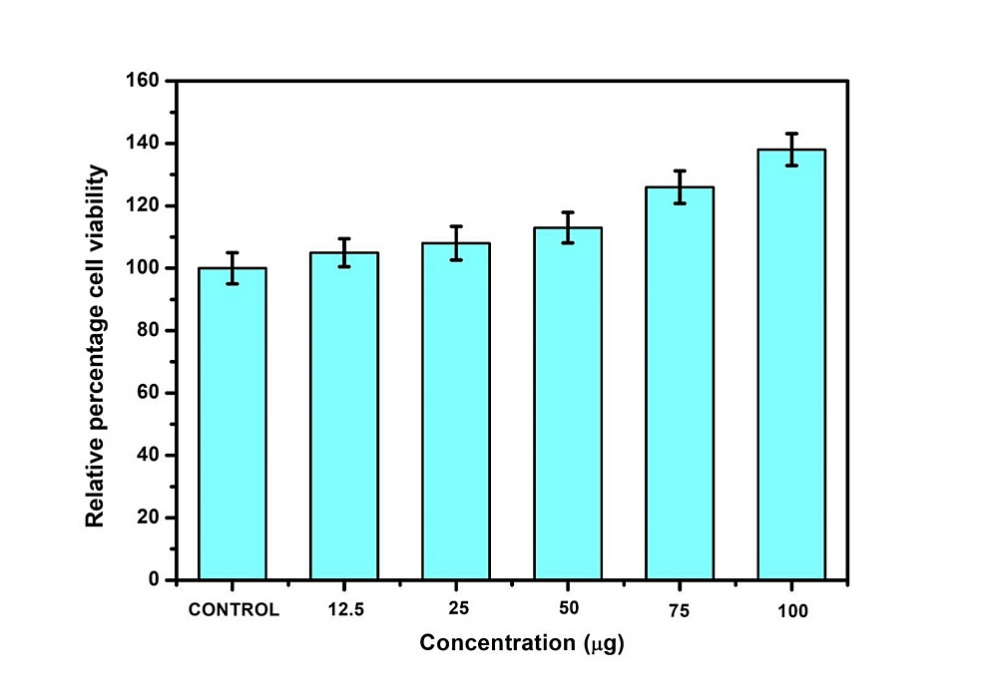
Relative percentage of cell viability across concentrations from 12.5 µg to 100 µg of Eu-MCaP Eu-MCaP: Europium-Monetite complex

The material's safety and effectiveness were evaluated across concentrations (12.5 µg to 100 µg), revealing increased cell viability and proliferation with higher percentages in the experimental group Eu-mCaP than the control group as shown in Figures 13A, 13B. Cells maintained their spindle shapes, emphasizing the excellent biocompatibility [13]. The relative percentage of cell viability observed between the control and Eu-mCaP across concentrations was tabulated in the bar graph as shown in Figure 14.

## Discussion

The adoption of green synthesis in material production stands as a pivotal and sustainable method for crafting biomaterials, presenting substantial advantages such as cost-effectiveness, reduced raw material consumption, and minimized toxicity [14]. *C. quadrangularis*, chosen for its role as a climber, possesses a rich array of properties including antioxidant, antibacterial, anti-hemorrhoidal, antifungal, anti-inflammatory, analgesic, and bone fracture healing attributes [15]. Employed in green synthesis, *C. quadrangularis* contributes to bone regeneration by stimulating the growth and specialization of mesenchymal stem cells, its effectiveness varying with dosage [14]. Additionally, it promotes the mineralization of the extracellular matrix and exhibits anti-inflammatory properties, creating a conducive environment for bone healing and regeneration, including enhanced angiogenesis to facilitate optimal distribution of oxygen and nutrients [13,16].

The incorporation of green synthesis in the production of *C. quadrangularis* may have played a role in shaping the Monetite structure, thereby augmenting the osteogenic activity of the innovative complex [16]. Europium, recognized as a soft and volatile rare earth element, is esteemed for its remarkable fluorescence capabilities, widely applied in cell imaging techniques. The novel Eu-MCaP, containing 0.01% mol of Eu, retains the inherent luminescent property of Europium, allowing for the assessment of the material's degradation ability when utilized in scaffolds or as an implant coating. Bioactive materials like calcium polyphosphate scaffolds, Hydroxyapatite (HAp), and bioactive glasses (BGs) can enhance their osteogenic capabilities through the addition of signaling ions in suitable amounts [14]. While Eu shares a structure similar to Ca, it boasts a larger ionic potential [16,17]. This enables it to regulate the expression of osteogenic markers such as ALP, COL1, OPN, and Runx2, influencing the release of Ca ions [18,19]. This modulation affects the conformation of HAp during new bone formation and impacts genes associated with osteogenesis. Moreover, Eu increases H_2_O_2_ synthesis, activating endothelial nitric oxide synthase (eNOS) through a PI3K-dependent pathway, ultimately promoting angiogenesis. The multifaceted properties of Eu encompass antibacterial efficacy, antitumor capabilities, and robust biocompatibility [20].

Monetite, classified as anhydrous dicalcium phosphate and a member of the calcium phosphate family, exhibits remarkable regenerative capacities, surpassing those of HAp-based graft materials in both volume generation and faster resorption, without converting to HAp [21,19]. Recognized as a degradable matrix, Monetite serves as an effective vehicle for delivering drug conjugates and fostering bone regeneration [22]. Idowu et al. investigation into Monetite's osteoinductive potential as a scaffold under a non-conditioned medium revealed that human mesenchymal cells maintained their typical physiological, morphological, and proliferative characteristics. The scaffold exhibited intrinsic osteoinductive properties, akin to control HAp [23].

Numerous studies have explored the doping of Europium onto calcium phosphate for drug delivery and creatinine estimation [24]. This study stands as a pioneer in synthesizing the Eu-MCaP and evaluating its osteogenic potential for effective bone regeneration. Considering the study's limitations, it is crucial to evaluate both the degradation profile and antimicrobial efficacy of the material. Additionally, we conducted an in vitro analysis to examine the properties of this groundbreaking Eu-MCaP. Future research will aim to further assess its antimicrobial abilities, mechanical characteristics, and hemocompatibility, and explore potential applications like incorporating it into scaffolds, utilizing it as a drug delivery platform, and applying it as coatings for implants. Subsequent investigations will be done to involve animal studies and human trials to comprehensively evaluate its potential.

## Conclusions

The innovative biomaterial, synthesized Europium-Monetite calcium phosphate complex, exhibits enhanced osteogenic potential with increased collagen and calcium staining, ALP activity, and robust biocompatibility. This biomaterial holds promise for various applications, including bone grafting, scaffold incorporation, and implant coatings. The novel Europium-Monetite calcium phosphate complex offers versatile applications in bone grafting, scaffold integration, and implant coatings, presenting a potential biomaterial with advanced osteogenic capabilities for effective bone regeneration. Future research should investigate the material's degradation profile, mechanical properties, antimicrobial properties, and broader applications in drug delivery and coating implants.
